# Systemic Inflammation in Cachexia – Is Tumor Cytokine Expression Profile the Culprit?

**DOI:** 10.3389/fimmu.2015.00629

**Published:** 2015-12-24

**Authors:** Emidio M. de Matos-Neto, Joanna D. C. C. Lima, Welbert O. de Pereira, Raquel G. Figuerêdo, Daniela M. dos R. Riccardi, Katrin Radloff, Rodrigo X. das Neves, Rodolfo G. Camargo, Linda F. Maximiano, Flávio Tokeshi, José P. Otoch, Romina Goldszmid, Niels O. S. Câmara, Giorgio Trinchieri, Paulo S. M. de Alcântara, Marília Seelaender

**Affiliations:** ^1^Cancer Metabolism Research Group, Faculdade de Medicina, Universidade de São Paulo, São Paulo, São Paulo, Brazil; ^2^Israelite Albert Einstein Institute, Israelite Albert Einstein Hospital, São Paulo, São Paulo, Brazil; ^3^Department of Clinical Surgery, Universidade de São Paulo, São Paulo, São Paulo, Brazil; ^4^Center for Cancer Research, NIH, Bethesda, MD, USA; ^5^Department of Immunology, Universidade de São Paulo, São Paulo, São Paulo, Brazil

**Keywords:** cancer cachexia, inflammatory cells, tumor-adipose tissue crosstalk macrophages

## Abstract

Cachexia affects about 80% of gastrointestinal cancer patients. This multifactorial syndrome resulting in involuntary and continuous weight loss is accompanied by systemic inflammation and immune cell infiltration in various tissues. Understanding the interactions among tumor, immune cells, and peripheral tissues could help attenuating systemic inflammation. Therefore, we investigated inflammation in the subcutaneous adipose tissue and in the tumor, in weight stable and cachectic cancer patients with same diagnosis, in order to establish correlations between tumor microenvironment and secretory pattern with adipose tissue and systemic inflammation. Infiltrating monocyte phenotypes of subcutaneous and tumor vascular-stromal fraction were identified by flow cytometry. Gene and protein expression of inflammatory and chemotactic factors was measured with qRT-PCR and Multiplex Magpix^®^ system, respectively. Subcutaneous vascular-stromal fraction exhibited no differences in regard to macrophage subtypes, while in the tumor, the percentage of M2 macrophages was decreased in the cachectic patients, in comparison to weight-stable counterparts. CCL3, CCL4, and IL-1β expression was higher in the adipose tissue and tumor tissue in the cachectic group. In both tissues, chemotactic factors were positively correlated with IL-1β. Furthermore, positive correlations were found for the content of chemoattractants and cytokines in the tumor and adipose tissue. The results strongly suggest that the crosstalk between the tumor and peripheral tissues is more pronounced in cachectic patients, compared to weight-stable patients with the same tumor diagnosis.

## Introduction

Cachexia is a multifactorial and multi-organ syndrome characterized by continuous and involuntary weight loss and by systemic inflammation (1, 2). This syndrome was described about 2000 years ago by Hippocrates and is a common feature of several diseases, such as chronic obstructive pulmonary disease, chronic heart failure, chronic infection, and cancer (3).

In cancer, cachexia is present in approximately 50% of all patients and in up to 80% of patients with advanced disease, reducing tolerance to treatment, therapeutic response, and quality of life and survival (4). Among 22–40% of all cancer deaths are directly caused by cachexia (5), and its incidence varies among the different types of cancer, being of around 80% in pancreas and gastrointestinal cancer patients, and of 60% in lung cancer patients (6).

An important feature of cachexia is chronic systemic inflammation and, paradoxically, immunosuppression (7). Mediators produced by both the tumor and the host induce intracellular changes directly associated with persistent inflammation (8). The sources of the inflammatory factors in cachexia are plenty, including tumor cells, tumor infiltrating cells along with peripheral tissue parenchymal cells and associated infiltrating cells (9). Thus, an intricate tumor–host interaction is established, promoting an imbalance that favors the pro-inflammatory over the anti-inflammatory status (10, 11).

Solid tumors often present infiltrating immune cells and release cytokines into surrounding tissues and into the bloodstream (12). The immune cells within tumor microenvironment consist of various phenotypes, among which myeloid-derived suppressor cells, dendritic cells, natural killers, T cells, and macrophages (13). The infiltrate contributes to tumor growth and also to microenvironment remodeling; while the release of cytokines into the bloodstream promotes tissue and organ functional impairment as a result of systemic inflammation (12). Studies with models have shown that the host’s tissues play a key role in sustaining systemic inflammation and inducting cachexia (14–17).

However, as far as we know, there are no reports in the literature comparing the cytokine secretory profile of tumors of cachectic and non-cachectic cancer patients matched for tumor type and stage. It is very possible that inflammatory factors secreted by the tumor are the culprit, eliciting secondary tissue inflammation, will as a consequence, fuel systemic inflammation. Argilés et al. review the large number of cytokines that might be responsible for the metabolic changes associated with cancer wasting (18). We have consistently found that WAT (white adipose tissue) is a contributor to systemic inflammation, as both adipocytes and infiltrating immune cells are capable of releasing cytokines in animal models of cachexia. Nevertheless, the mechanisms that trigger adipose inflammation in cancer cachexia are not fully elucidated. We hypothesize that differences in tumor microenvironment and secretion pattern in patients with the same diagnosis and tumor stage could be associated with the presence or absence of cachexia-related peripheral tissue inflammation.

The aim of the present study was therefore, to examine the secretory profile of tumors of cachectic and non-cachectic patients with matched tumor diagnosis and relate to the results with local white adipose tissue and systemic inflammation.

## Materials and Methods

### Subjects

Twenty-three cancer patients (60.53 ± 13.08 years old) participated in the study. The study was approved by the University of São Paulo Biomedical Sciences Institute Ethics Committee (1004/CEP) and by the University Hospital Ethics Committee (CEP-HU/USP: 752/07) in accordance to the *Declaration of Helsinki* (2013). All participants signed an informed consent prior to engaging in the study. The inclusion criteria were: not having received anticancer or continuous anti-inflammatory treatment and willingness to participate. The exclusion criteria were: liver failure, renal failure, AIDS, inflammatory diseases of the bowel, and autoimmune disorders. Patient group division was based on the criteria proposed by Evans et al. (19). Characteristics of the subjects are summarized in Table 4.

### Realtime PCR

Total RNA was isolated from samples, with Trizol^®^ reagent (Invitrogen, Carlsbad, CA, USA) following the manufacturer’s recommendations, and then homogenized. RNA concentrations were determined by measuring the absorbance in 260/280 nm in Synergy H1 Multi-Mode Reader (Thermo Fisher Scientific Inc., Waltham, MA, USA). Complementary DNA synthesis was carried out using the high capacity cDNA reverse transcription kit (Life Technologies, Grand Island, NY, USA), which consisted of an assay mix containing 1 μg total RNA, 2 μL 10× RT Buffer, 0.8 μL 25× dNTP mix (100 mM), 2 μL 10× Random primers, 1 μL MultiScribe™ Reverse Transcriptase, and 4.2 μL of nuclease-free water in a final volume of 20 μL. The thermal cycler conditions were: 25°C for 10 min, then 37°C for 120 min followed by 85°C for 5 min. Then, 20 ng of cDNA was mixed with 2× SYBR Green fast PCR master mix – and primers (Table 1) (Life Technologies, Grand Island, NY, USA) – in a final volume of 10 μL for qPCR, performed in the Quantstudio 12K Real Time Systems (Life Technologies, Grand Island, NY, USA). The mRNA levels were determined by the comparative Ct method. For each sample, a ΔCt value was obtained by subtracting RPL-27 or HPRT1 gene values from those of the gene of interest. The average ΔCt value of the control group was then subtracted from the sample to derive a −ΔΔCt value. The expression of each gene was evaluated by 2−ΔΔCt, according to Livak and Schmittgen (20).

**Table 1:** **List of primers**.

Gene (species)	Sequence 5**′→**3**′**
*CCL-2* (*Homo sapiens*) (NM 002982.3)	Fw: TCA GCC AGA TGC AAT CAA TG
	Rev: ACA CTT GCT GCT GGT GAT TCT
*IL-1*β** (*Homo sapiens*) (NM 000576.2)	Fw: AGC CAA TCT TCA TTG CTC AAG T
	Rev: AGT CAT CCT CAT TGC CAC TGT
*IL-6* (*Homo sapiens*) (*NM* 000600.3)	Fw: CAG CCC TGA GAA AGG AGA CAT
	Rev: AGC CAT CTT TGG AAG GTT CA
*IFN-*γ** (*Homo sapiens*) (NM 000619.2)	Fw: TGG AAA GAG GAG AGT GAC AGA A
	Rev: TTG GAT GCT CTG GTC ATC TTT A
*TNF-*α (*Homo sapiens*) (NM 000594.3)	Fw: CTC TCT CCC CTG GAA AGG AC
	Rev: ATC ACT CCA AAG TGC AGC AG
*IL- 10* (*Homo sapiens*) (NM 000572.2)	Fw: TGTCATCGATTTCTTCCCTGT
	Rev: TGC CTT TCT CTT GGA GCT TAT T
*RPL-27*(*Homo sapiens*) (NM 000988.3)	Fw: CCG AAA TGG GCA AGT TCA T
	Rev: CCA TCA TCA ATG TTC TTC ACG A
*IL-8* (*Homo sapiens*) (NM 000584.3)	Fw: AGC TCT GTG TGA AGG TGA T
	Rev: TTT GGG GTG GAA AGG TTT G
*ZAG* (*Homo sapiens*) (NM 001185.3)	Fw: CCA GGA GAA CCA AGA TGG TC
	Rev: CTG CTT CCA ATC CTC CAT TC
*PIF* (*Homo sapiens*) (NM 005268627.1)	Fw: AGG AAG CAG AGA TCC AGC CT
	Rev: GGC TCC TTT ACC CAC GCT TT
*HPRT1*(*Homo sapiens*) (NM 000194.2)	Fw: TGG CGT CGT GAT TAG TGA TG
	Rev: CTT GAG CAC ACA GAG GGC TA

### Multiplex Analysis of Sample Protein Content

Samples of the tumor and subcutaneous adipose tissue from the experimental groups were incubated with the mixture of Magplex microspheres and covered with the specific antibodies for 2 h. The detection of target antigens bound to the microspheres was performed with a mixture of biotinylated capture antibodies after incubation for 1 h followed by incubation with streptavidin labeled with phycoerithrin for 30 min. The microspheres were then analyzed with the phycoerithrin Magpix^®^ instrument (Life Technologies, Grand Island, NY, USA). Each cytokine value was corrected to total protein concentration. The table below describes all analyzed cytokines (Table 2).

**Table 2:** **Cytokine analysis**.

Cytokine	Abbreviation
Tumor necrosis factor alpha	TNF-α
Tumor necrosis factor beta	TNF-β
Interleukin 6	IL-6
Interleukin 7	IL-7
Interleukin 10	IL-10
Interleukin 13	IL-13
Interferon alpha	IFN-α
Interferon gamma	IFN-γ
Interferon gamma-induced protein 10	IP-10
Monocyte chemotactic protein1	MCP1/CCL2
Macrophage inflammatory protein-1α	MIP-1α/CCL3
Macrophage inflammatory protein-1β	MIP-1β/CCL4
Chemokine(C–C motif) ligand 5	RANTES/CCL5

### Immunophenotyping by Flow Cytometry

#### Preparation of Adipose Tissue and Tumor Cells for Flow Cytometry

Fractions of subcutaneous adipose tissue and tumor were obtained, any lymph nodes were carefully removed, and the tissues were placed in either DMEM (Dulbecco’s Modified Eagle Medium) or HBSS (Hank’s Balanced Salt Solution). The tissue fragments were then digested for 40 min at 37°C in these culture media containing collagenase type I (280 U/ml) (Sigma Aldrich) under agitation. The samples were filtered through fine plastic mesh and washed with respective media.

Finally, cells of vascular-stromal fraction were separated by centrifugation at 500 *g* for 5 min. The cells of the stromal-vascular fraction of adipose tissue were resuspended and washed twice with culture medium and centrifuged again at 500 *g*, for 5 min. The cells were resuspended in 500 μL of FBS and dimethyl sulfoxide (DMSO) and stored in liquid nitrogen until processing for flow cytometry.

### Cell Surface Antigens for Flow Cytometry

The samples were rapidly thawed in a water bath at 37°C, washed with culture medium, and pelleted at 600 g for 10 min at 4°C. Compensation of the flow cytometer (FACSCanto II – BD Biosciences) was performed with compensating beads and then the gates were determined for the analysis of cell populations of interest (Figure S1 in Supplementary Material).

The fluorochrome conjugated antibodies (listed in Table 3) of the macrophage panels were added to the samples, and these were incubated for 30 min at 4°C, in the dark. The labeled cells were washed, centrifuged 400 *g* for 5 min, resuspended in 500 μL of DMEM, and detected by BD FACSCanto^TM^ II cytometer.

**Table 3:** **Panels of fluorochrome-conjugated antibodies for flow cytometry**.

Panel	Antibody	Fluorochrome	Catalog no.
Macrophages (M1 and M2)	CD45	FITC	555482
	CD206	PE	555954
	CD14	PERCP-Cy5.5	562692
	CXCR4	PE-Cy7	560669
	CD86	APC	555660
	CD11b	APC-Cy7	557657
	CCR7	BV421	562555

### Statistical Methods

Data are expressed as mean ± SE or median [first quartile; third quartile]. First, a Gaussian distributions test was employed for all samples (D’Agostino-pearson omnibus test, Shapiro–Wilk test, Kolmogorov–Smirnov Test). Student’s *t*-test or Mann–Whitney test with multiple comparisons was employed for parametric and non-parametric data, respectively. The significance level was set at *p* < 0.05. Graphpad Prism 5.0 was adopted for the analysis. All statistical procedures were performed with the assistance of the Institute of Biomedical Sciences/University of Sao Paulo, under the supervision of Ms. Rosana Duarte Prisco.

## Results

### General Characteristics of Patients

The general characteristics of patients are illustrated in Table 4. No statistical differences were found in regard to age and height between the groups. Body mass in the 12 months before engagement in the study, as informed by the patients at moment of the recruitment interview, showed no statistical differences between groups, while baseline body mass of the cachectic cancer group was lower (in average 11%), when compared with the weight-stable cancer group, although not statistically significant (*p* = 0.07). When comparing the difference between previously informed body mass and current body mass, marked weight loss (both in terms of absolute and relative weight) was found for CC, in relation to the weight-stable cancer (WSC) group, in accordance with the proposed by Evans et al. (19) (weight loss >5% over past 6 months – in absence of simple starvation). The body mass index (kg/m^2^) of CC, although greater than 20 kg/m^2^ (considered the cutoff point for cachexia), was significantly lower than that of WSC. C-reactive protein, albumin, hemoglobin, and IL-6, biochemical markers of cachexia, were also evaluated. CRP plasma content – the most widely accepted index of systemic inflammation – was higher in CC than in WSC (*p* = 0.0026). Similarly, plasma IL-6 levels were significantly higher in cachectic cancer patients (CC) (*p* = 0.0119). Additionally, serum hemoglobin levels of CC were consistently lower when compared with WSC (*p* = 0.0064). Serum albumin levels were not significantly different between groups (*p* = 0.316).

**Table 4:** **General characteristic of patients**.

	WSC (weight-stable cancer)	CC (cachectic cancer)	*p*
*N*	17	19	
Male/female (*n*)	10/7	12/7	
Age (years)	59.2 ± 3.69	61.7 ± 2.55	0.582
Height (m)	1.65 ± 0.024	1.65 ± 0.018	0.936
Previous body mass as informed (kg)	74.1 ± 3.13	72.3 ± 3.21	0.695
Current body mass (kg)	70.5 ± 3.17	62.5 ± 2.86	0.07
Weight loss (kg)	0.00 [0.00; 6.50]	10.00 [5.00; 13.00]^a^	0.0009
Weight loss (%)	0.00 [0.00; 9.00]	12.0 [8.00; 16.0]^a^	0.0006
BMI (kg/m^2^)	25.9 ± 1.04	22.8 ± 0.76^a^	0.0195
Tumor stage (*n*)			
I-II	4	7	
III-IV	13	12	
CRP (mg/L)	3.95 [0.90; 8.03]	11.7 [7.15; 13.5]^a^	0.0026
Albumin (g/dL)	4.32 ± 0.18	4.04 ± 0.21	0.316
Hemoglobin (g/dL)	13.4 ± 0.50	11.2 ± 0.57^a^	0.0064
IL-6 (pg/mL)	2.67 ± 0.65	9.84 ± 2.02^a^	0.0119

*Data expressed as mean ± SE or as median [first quartile; third quartile]*.

*^a^Significant difference CC vs. WSC group*.

*BMI, body mass index; CRP, C-reactive protein; IL-6, interleukin 6*.

### Tumor Gene Expression Analysis

**Figure 1:**
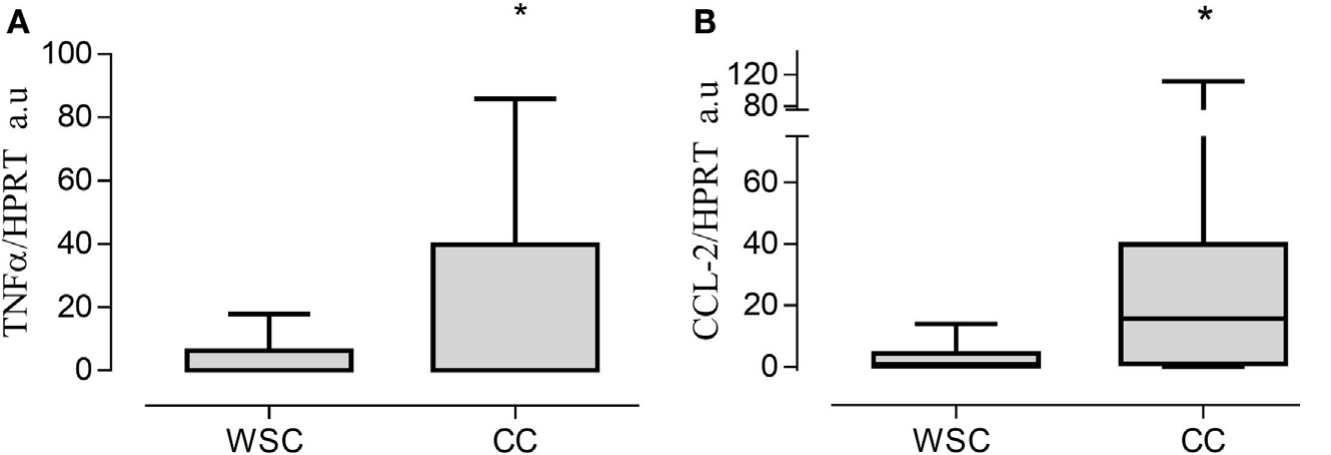
**Gene expression in tumor tissue**. Data expressed as mean ± SE or as median [first quartile; third quartile]. *Significant difference between WSC vs. CC. Expression of target genes was normalized to the reference HPRT1. TNF-α, tumor necrosis factor α **(A)**; CCL2, chemokine (C–C motif) ligand 2 **(B)**; Arbitrary units, AU. WSC (*n* = 10) and CC (*n* = 14).

Gene expression of the pro-inflammatory cytokines TNF-α and CCL2 in the tumor were increased in CC compared to WSC, *p* = 0.020 and *p* = 0.0354, respectively (Figures 1A–B). No statistically significant difference in mRNA concentration of VEGF (angiogenesis factor), IL-6, IL-1β, IFN-γ, PIF, ZAG, IL-10, between WSC and CC could be detected, as shown in Table 5.

**Table 5:** **Tumor gene expression of cytokines and cachexia-related factors (AU)**.

qRT-PCR (A.U)	WSC (weight-stable cancer)	CC (cachectic cancer)	*p*
VEGF	1.275 [0.446; 8.270]	0.557 [0.069; 3.28]	0.410
IL-6	1.395 [0.368; 2.509]	1.163 [0.537; 8.330]	0.683
IL1-β	2.545 [0.430; 16.07]	0.791 [0.185; 7.893]	0.524
IFN-γ	1.317 [0.313; 5.095]	27.65 [0.420; 80.16]	0.151
PIF	0.711 [0.154; 9.012]	9.706 [0.023; 101.1]	0.571
ZAG	2.029 [0.374; 3.501]	0.716 [0.369; 2.766]	0.497
IL-10	0.728 [0.152; 10.93]	34.12 [0.141; 54.02]	0.398

*Data expressed as median [first quartile; third quartile]. Target gene expression was normalized to the housekeeping gene hypoxanthine-guanine phosphoribosyltransferase (HPRT-1)*.

*Arbitrary units (AU). WSC (*n* = 10); CC (*n* = 14)*.

### Subcutaneous Adipose Tissue Gene Expression Analysis

As previously described, we found that gene expression of TNF-α, IL-1β, and MCP-1/CCL2 were significantly higher in cachectic cancer patients when compared with WSC. IL-6 and IFN-γ gene expression showed no differences among the groups.

### Tumor Protein Expression Analysis

**Figure 2:**
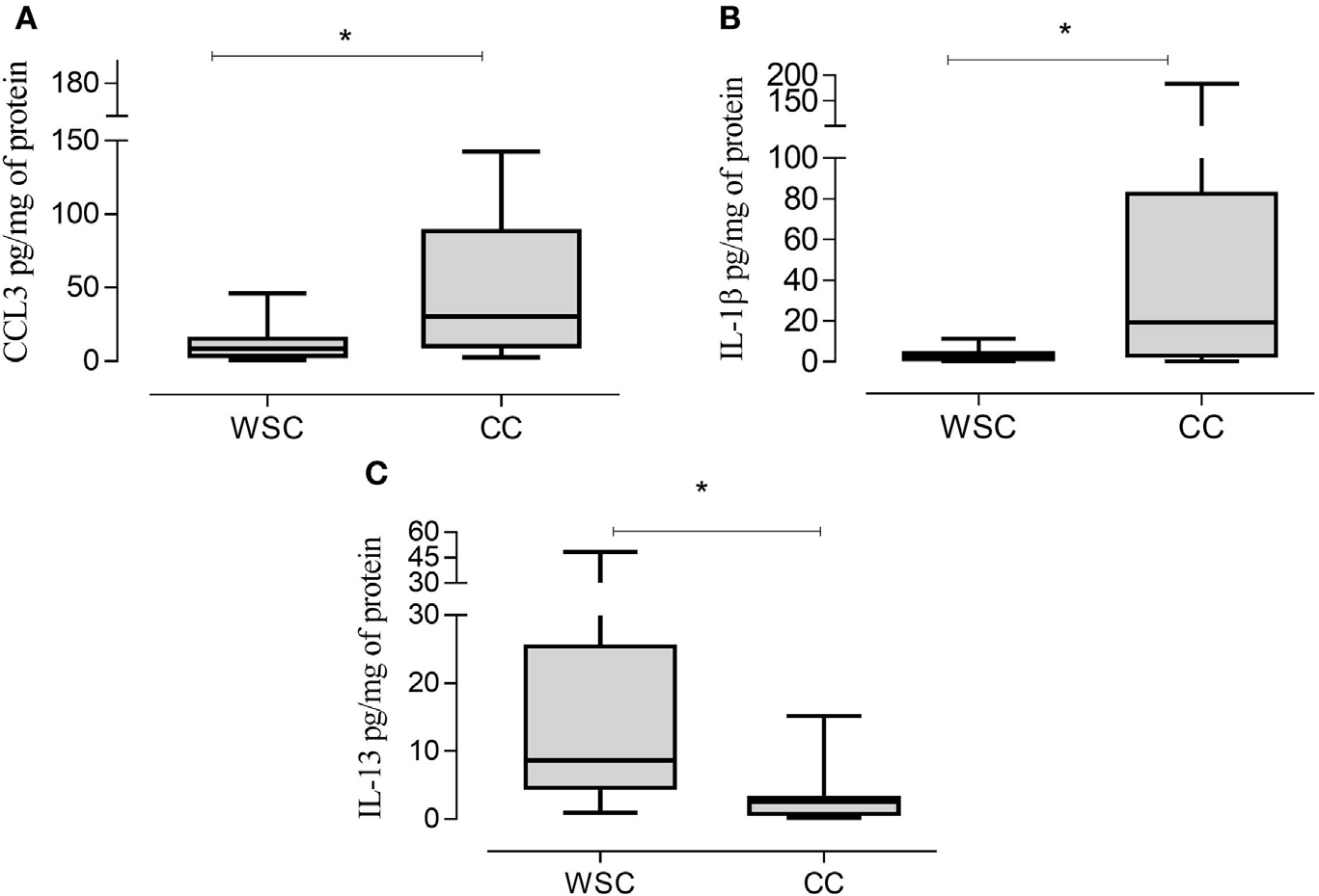
**CCL3, IL-1β, and IL-13 protein expression in tumor samples**. Data expressed as median [first quartile; third quartile]. *Significant difference between WSC vs. CC. CCL3, chemokine (C–C motif) ligand 3 **(A)**; IL-1β, interleukin 1β **(B)**; IL-13, interleukin 13 **(C)**. WSC (*n* = 11) and CC (*n* = 12).

Protein expression of chemoattractant factors in tumor tissue CCL [(chemokine (C–C motif) ligand)]-2, CCL4, CCL5 was not significantly different between the groups as shown in Table 6. However, CCL3, also known as macrophage inflammatory protein 1 alpha, was higher in CC in relation to WSC (*p* = 0.043) (Figure 2A).

**Table 6:** **Inflammatory factors in tumor samples**.

Pico gram per milligram of total protein	WSC (weight-stable cancer)	CC (cachectic cancer)	*p*
CCL2	230.5 [96.08; 373.1]	261.89 [124.1; 546.4]	0.431
CCL4	9.32 [3.92; 13.41]	16.62 [6.77; 55.84]	0.060
CCL5	649 ± 99.69	977.8 ± 272.2	0.306
IFN-α	20.34 [5.65; 51.66]	10.95 [7.76; 52.70]	0.791
IL-10	0.363 [0.22; 1.58]	0.441 [0.16; 2.42]	0.725
IL-6	1.034 [0.245; 1.92]	2.097 [0.724; 8.33]	0.194
IP-10	243.7 [151.0; 352.2]	1263 [179.8; 2822]	0.092
TNF-α	0.352 [0.202; 0.908]	0.724 [0.339; 1.55]	0.169
TNF-β	2.306 ± 0.567	2.435 ± 0.601	0.878

*Data expressed as mean ± SE or as median [first quartile; third quartile], *p* = significance of Mann–Whitney test. Cytokine concentration was normalized to total protein. WSC (*n* = 11); CC (*n* = 12)*.

The protein concentrations of different pro- and anti-inflammatory cytokines and cachexia-related factors in cachectic and non-cachectic cancer are shown in Table 6. Among the pro-inflammatory cytokines, IL-1β was increased in CC compared to WSC (*p* = 0.041) (Figure 2B). Protein concentration of IP-10, a chemokine secreted by interferon stimulated cells was not significantly different but showed a tendency to be significantly higher in CC (*p* = 0.092). Other inflammatory cytokines such as IFN-γ and IL-6 were not significantly different between the groups. Members of the tumor necrosis factor family TNF-α and TNF-β were also not statistically different in CC compared to WSC. The protein concentration of anti-inflammatory interleukins IL-10 was not different (*p* = 0.9652) between groups, yet that IL-13 (*p* = 0.007) was lower in CC in compared WSC (Figure 2C).

### Subcutaneous Adipose Tissue Protein Expression Analysis

**Figure 3:**
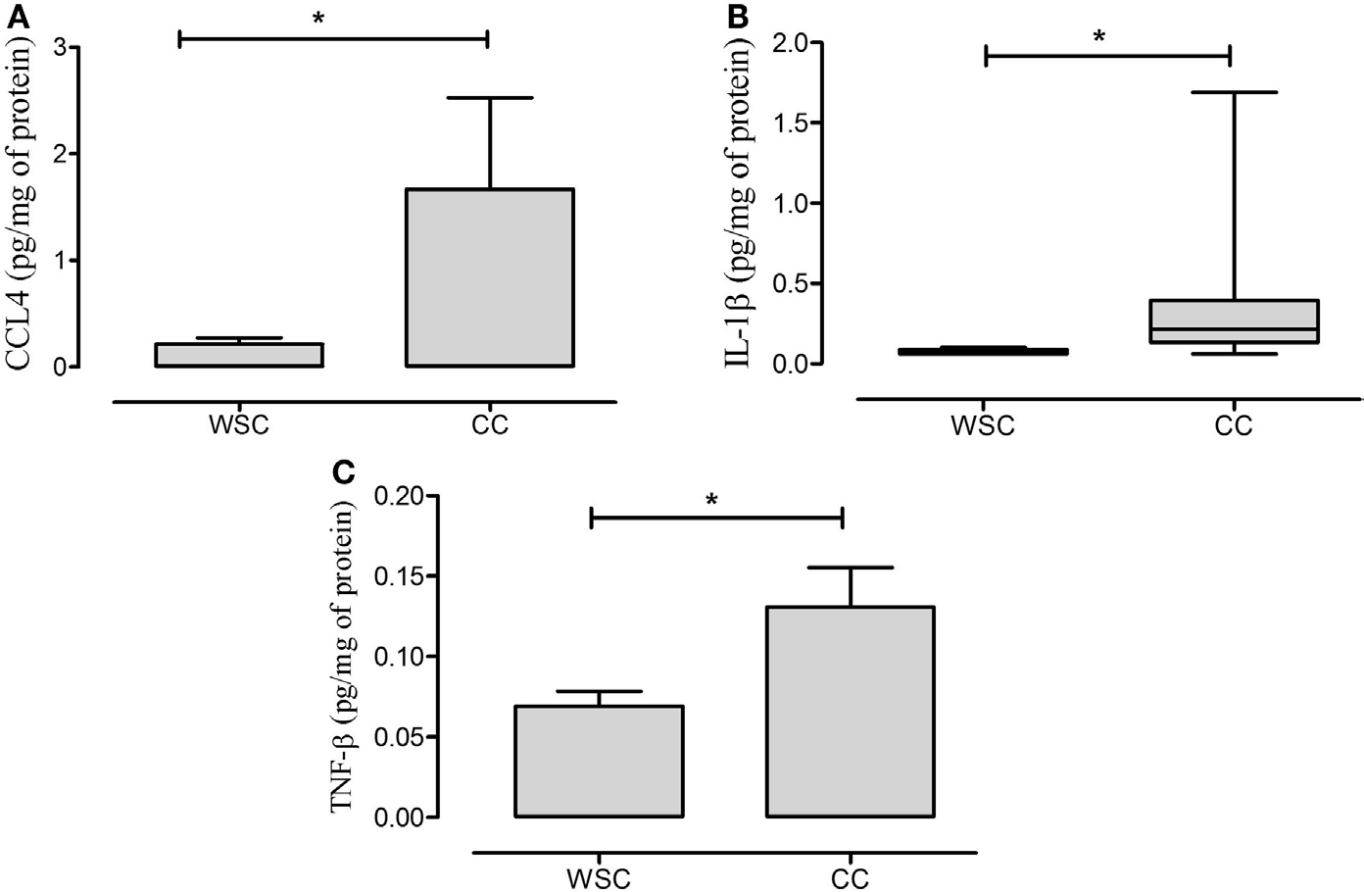
**CCL4, IL-1β, and TNF-β protein expression in subcutaneous adipose tissue**. Data expressed as mean ± SE. *Significant difference CC vs. WSC group. CCL4, chemokine (C–C motif) ligand 4 **(A)**; IL-1β, interleukin 1β **(B)**; TNF-β, tumor necrosis factor β **(C)**. WSC (*n* = 11) and CC (*n* = 12).

Data of protein expression of chemoattraction factors are shown in Table 7. We found no statistical difference for CCL2, CCL3 and CCL5 in subcutaneous adipose tissue (Table 7). CCL4 protein expression was higher in CC, when compared with WSC (Figure 3A).

**Table 7:** **Inflammatory factors in the subcutaneous adipose tissue**.

Pico gram per milligram of total protein	WSC (weight-stable cancer)	CC (cachectic cancer)	*p*
CCL2	38.0 ± 7.20	20.3 ± 5.26	0.0646
CCL3	13.0 [4.06; 59.4]	3.38 [0.010; 68.6]	0.3725
CCL5	157 ± 31.0	121 ± 30.6	0.4219
IFN-α	0.210 [0.135; 3.68]	2.12 [0.228; 4.73]	0.2883
IL-10	0.070 [0.060; 0.123]	0.100 [0.060; 0.330]	0.2275
IL-13	0.190 [0.110; 1.63]	0.500 [0.170; 0.680]	0.6480
IL-6	0.0711 ± 0.004	0.101 ± 0.024	0.2668
IP-10	9.19 ± 2.42	3.63 ± 0.919	0.0522
TNF-α	0.050 [0.040; 0.0525]	0.055 [0.030; 0.103]	0.5140

*Data expressed as mean ± SE or as median [first quartile; third quartile], *p* = significance of Mann–Whitney test. Cytokine concentration was normalized to total protein. WSC (*n* = 11); CC (*n* = 12)*.

Anti- as well as pro-inflammatory cytokines (IFN-α, IL-10, IL-13, IL-6, IP-10, and TNF-α) did not exhibit differences between the two studied groups (Table 7). The pro-inflammatory IL-1β and TNF-β cytokines protein expression presented higher levels in CC in relation to WSC (Figures 3B,C, respectively).

### Immunophenotyping by Cytometry

**Figure 4:**
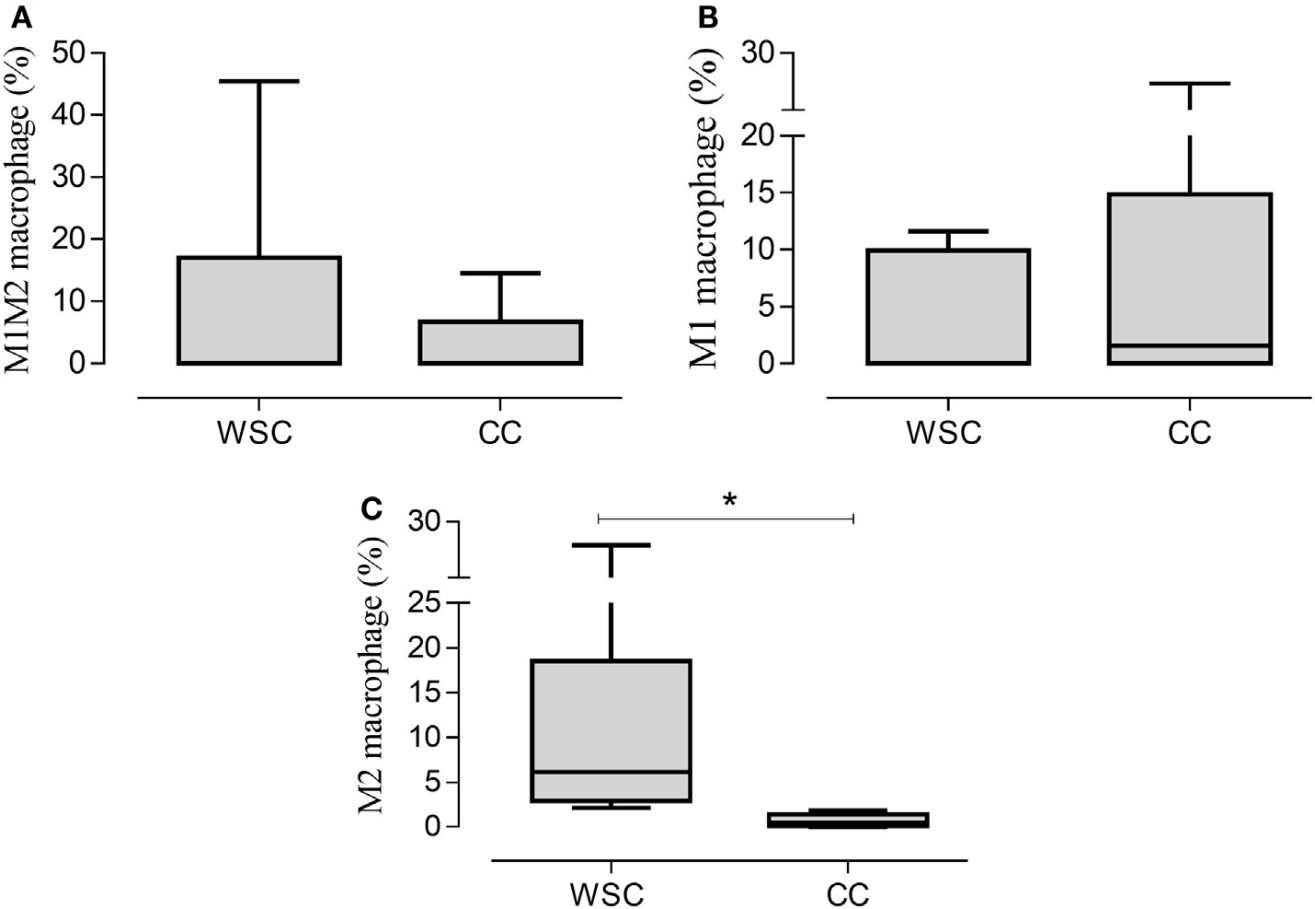
**Percentage of the phenotypes of macrophage populations in the tumor microenvironment**. Data expressed as median [first quartile; third quartile] or median ± SE. *Significant difference between WSC and CC. Tumor samples WSC and CC (*n* = 5). M1M2 macrophage **(A)**; M1 macrophage **(B)**; M2 macrophage **(C)**.

The characterization of the different phenotypes within the total population of infiltrating macrophages in the tumor microenvironment is shown in Figure 4. The incidence of macrophages with anti-inflammatory profile (M2 macrophages – CD11b CD14++ CXCR4+) was significantly lower in CC, compared to WSC (*p* = 0.007). Macrophages with inflammatory profile (M1 macrophages – CD11b+ CD14++ CCR7+) were found in similar numbers in the tumors of both groups.

**Figure 5:**
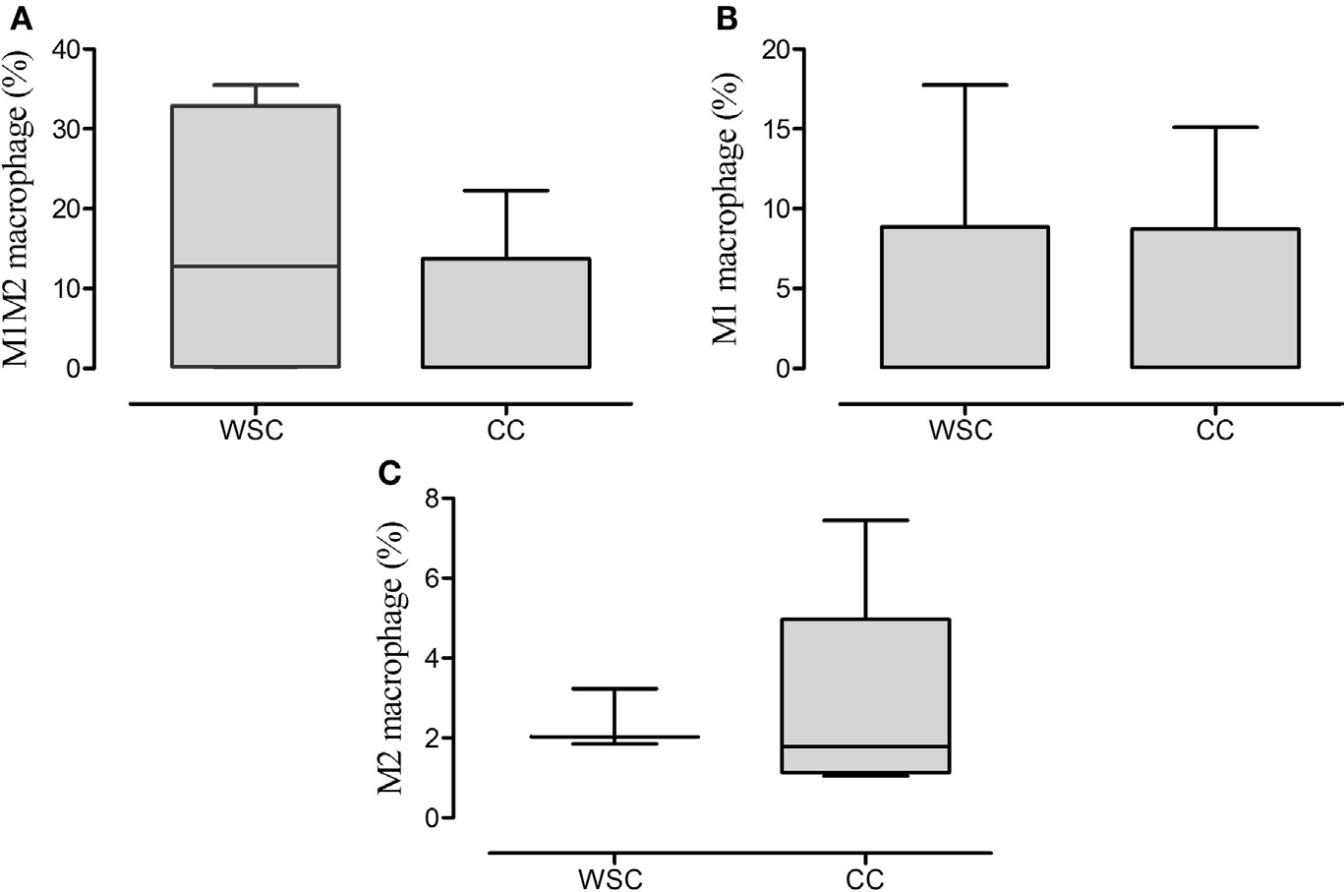
**Percentage of the phenotypes of macrophage in subcutaneous adipose tissue**. Data expressed as median [first quartile; third quartile]. Stromal-vascular fraction of subcutaneous adipose tissue: WSC (*n* = 4) and CC (*n* = 5). M1M2 macrophage **(A)**; M1 macrophage **(B)**; M2 macrophage **(C)**.

The analysis of the stromal-vascular fraction of the subcutaneous adipose tissue yielded no statistic difference in concern to M1M2 macrophage (CD11b CD14^++^ CCR7^+^ CXCR4^+^), M1 macrophage (CD11b^+^ CD14^++^ CCR7^+^) and M2 macrophage (CD11b CD14^++^CXCR4^+^) population percentage (Figures 5A–C, respectively).

### Correlations Analysis

**Figure 6:**
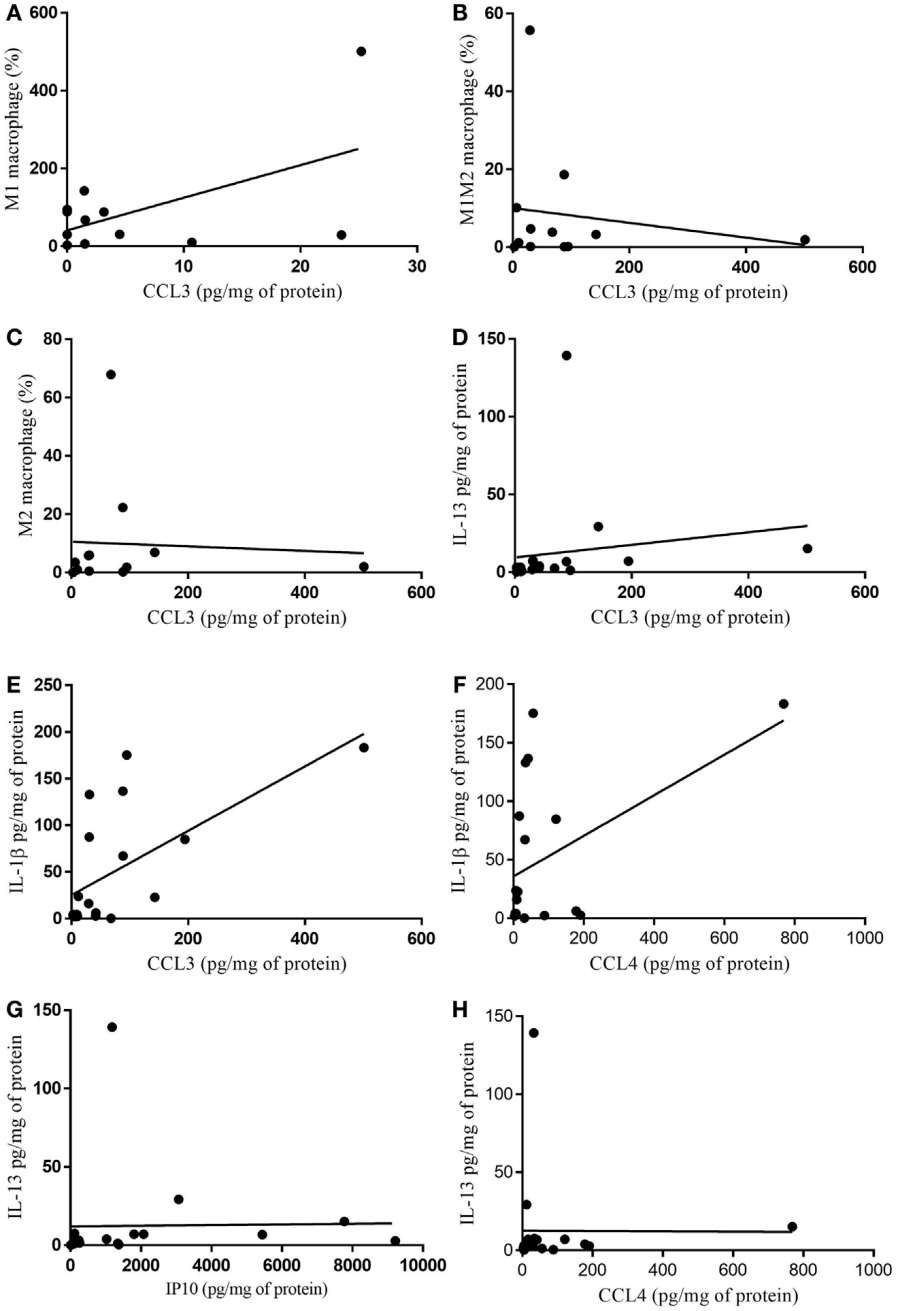
**Correlation of cytokine protein expression and % of infiltrating immune cells in tumor**. **(A)** CCL3/M1 macrophage (%) *p* = 0.938; **(B)** CCL3/M1M2 macrophage (%) *p* = 0.956; **(C)** CCL3/M2 macrophage (%) *p* = 0.342; **(D)** CCL3/IL-13 *p* = 0.0089; **(E)** CCL3/IL-1β *p* = 0.0059; **(F)** CCL4/IL-1β *p* = 0.089; **(G)** IP10/IL-13 *p* = 0.057; **(H)** CCL4/IL-13 *p* = 0.147.

Non-parametric correlation (Spearman) analysis between chemokine (C–C motif) ligand (CCL)-3 and CCL-4 with the protein expression of the cytokine anti-inflammatory cytokine IL-13 in the tumor of cachectic patients was found to be significant (*p* = 0.0089); while the relationship between CCL4 and IL-13 (*p* = 0.147) was not (Figures 6D,H). Analysis of correlation of CCL3 with the protein expression of the inflammatory cytokine IL-1B showed positive relationship (CCL3/IL-1β) (*p* = 0.0059) (Figure 6E). Whether the CCL4/IL-1β correlation (*p* = 0.0897) (Figure 6F) nor of CCL3 with %macrophages were found to be significant (Figures 6A–C).

**Figure 7:**
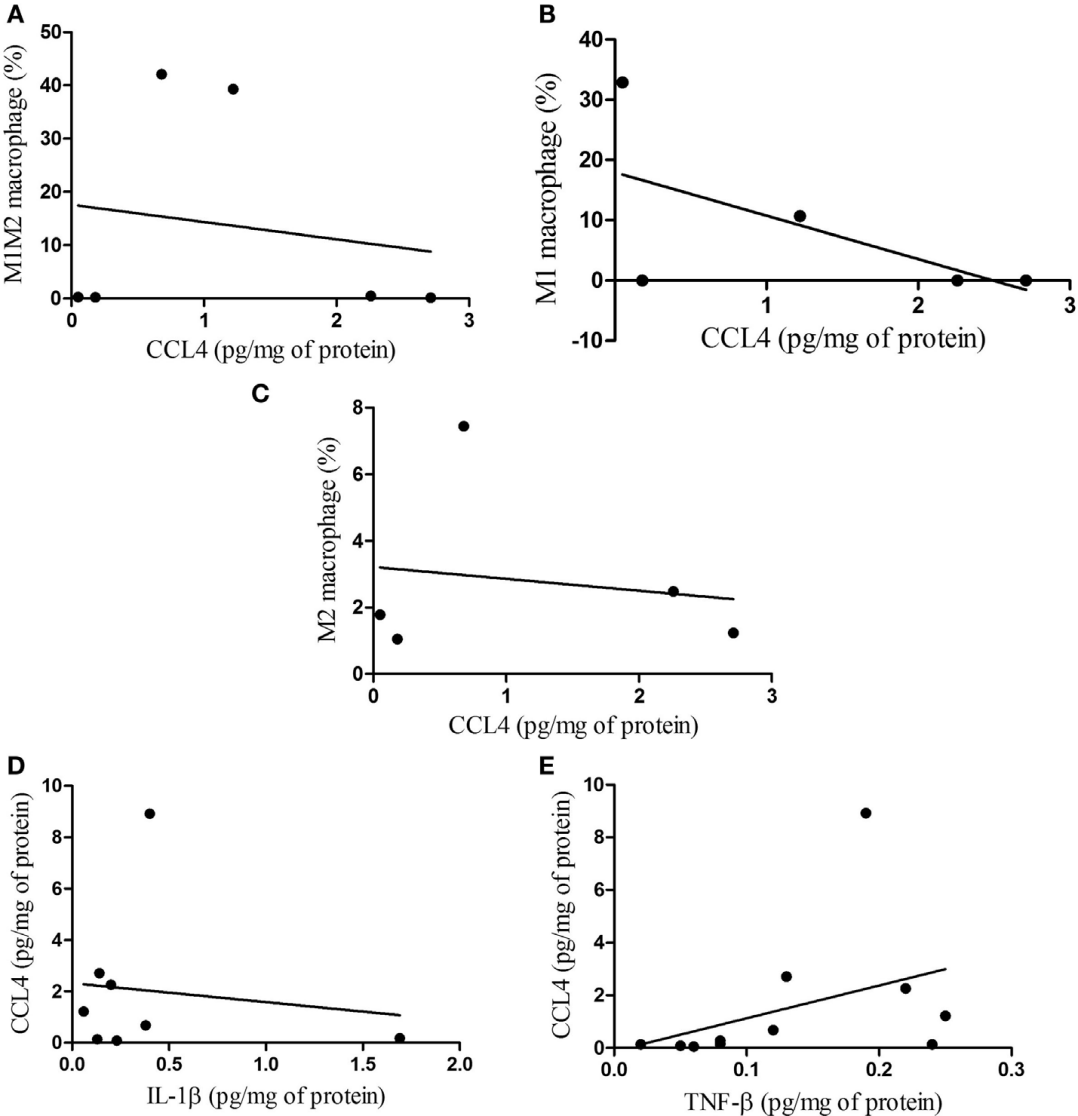
**Correlations between macrophage phenotypes and CCL4 protein, and between CCL4 and IL-1β, TNF-β in subcutaneous adipose tissue**. **(A)** M1M2/CCL4, *p* = 0.787; **(B)** M1/CCL4, *p* = 0.321; **(C)** M2/CCL4, *p* = 0.790 and correlations between CCL4 protein and IL-1β, TNF-β **(D)** CCL4/IL-1β, *p* = 0.955; **(E)** CCL4/TNF-β, *p* = 0.041.

When non-parametric correlation (Spearman) analysis was carried out in regard to macrophages and CCL4 in the subcutaneous adipose tissue, no statistical correlations were observed for M1M2 macrophages not for M1 macrophages, or M2 macrophages (Figures 7A–C, respectively). Furthermore, non-parametric correlation for CCL4 and IL-1β was found not to be significant, whereas that between CCL4 and TNF-β was significant (Figures 7D,E, respectively).

**Figure 8:**
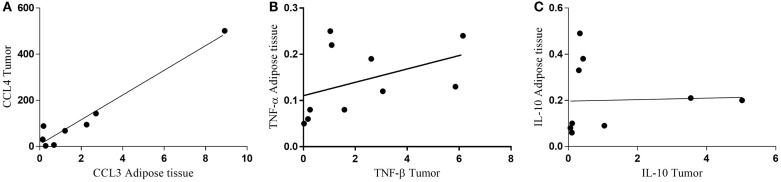
**Correlation between protein expression of inflammatory factors in subcutaneous adipose tissue and tumor**. **(A)** CCL4 tumor/CCL3 adipose tissue; **(B)** TNF-α adipose tissue/TNF-β tumor; **(C)** IL-10 adipose tissue/IL-10 tumor.

Finally, we performed non-parametric correlation (Spearman) analysis for CCL4 in the subcutaneous adipose tissue and for CCL3 in the tumor, having found a statistically significant positive correlation (*p* = 0.0448) only for the cachectic patients (Figure 8A). When the relationship of TNF-α in the subcutaneous adipose tissue and TNF-β in the tumor was analyzed, no statistical significance was found for CC (*p* = 0.0892) (Figure 8B). A tendency for positive correlation between IL-10 in subcutaneous adipose tissue and in the tumor (*p* = 0.0978) (Figure 8C).

## Discussion

Cancer cachexia remains a major health problem worldwide as prevalence of cancer is on the rise. This syndrome is frequently undiagnosed and rarely treated, resulting in compromising of treatment and shortened survival (1, 10). Weight loss is the most visible feature of cachexia, yet some early metabolic and inflammatory changes precede the establishment of the most evident symptoms. The cachectic patients in the study, beyond presenting severe weight loss in the previous 6 months, exhibited systemic inflammation and anemia (CRP >5.0 mg/L, IL-6 >4 pg/mL, Hb <12 g/dL), in accordance to that proposed by Evans et al. (19), but no alterations of circulating albumin levels.

Cachexia-associated inflammation is the result of many alterations acting in concert, among which, the secretion of inflammation-promoting factors by the tumor itself. This, on the other hand, may elicit tissue and organ local sustained inflammation, in a vicious cycle. One such mechanism has been proposed to exist in cancer patients (2, 21).

Obesity research has provided solid evidence that the adipose tissue is an important player in the onset and maintenance of systemic inflammation (22). Indeed, the adipose tissue produces numerous bioactive molecules as TNF-α, IL-1β, IL-6, CCL2, to cite a few; all of which are able to act in an autocrine, paracrine, and endocrine manner, hence reaching the blood stream and promoting the crosstalk with other tissues (23).

In cancer cachexia, we have previously shown evidence that the white adipose tissue is a potential contributor for systemic inflammation, as it suffers comprehensive rearrangement and immune cell infiltration, in association with robustly increased secretion of inflammatory factors (15, 24–26). Furthermore, the white adipose tissue of Walker 256 tumor-bearing rats was found to be infiltrated with monocytes (24), and we recently reported immune infiltration in cachectic cancer patients (25).

In another recent study employing the animal model of cachexia, we found up-regulation of IL-1β expression and activation of NF-κB and of the inflammasome pathways in adipocytes, and evidence of a major contribution of the vascular-stromal fraction of the retroperitoneal adipose tissue to tissue inflammation (26). In the current study, we have similarly found a population of infiltrated macrophages in the subcutaneous adipose tissue of cachectic patients, despite lack of statistical difference between the cachectic and non-cachectic groups in regard to the predominance of different macrophage phenotypes (M1M2, M1, and M2).

We also previously reported that NF-κBp65 gene expression is increased in the subcutaneous white adipose tissue of cachectic cancer patients, concomitantly to up-regulation of its inflammatory target genes IL-1β, TNF-α, CCL2/MCP-1, and IκB-α. Haugen et al. also found alterations in gene expression, including of TNF-α and CCL2, in the intra-abdominal adipose tissue, which was associated with reduced fat mass in patients with pancreatic cancer (27, 28).

To our knowledge, we are the first to show that the subcutaneous adipose tissue of cachectic patients presents higher CCL4 protein content in relation to WSC with matched tumor diagnosis. Increased CCL4 gene expression was found by Wu et al. (29) in the adipose tissue of obese mice, with concomitant augment of the number infiltrating leukocytes. In the present study, increased IL-1β and TNF-β protein expression was also detected, corroborating our previous findings (27).

However, what are the stimuli inducing adipose inflammation? The group of Michael Tisdale has approached, in several studies (10, 30–32), the role of tumor-derived factors in the onset of cachexia. Therefore, the main aim of the present study was to address the eventual differences in tumor microenvironment in cachectic and weight-stable cancer patients that could be possibly linked to the presence of cachexia. For that purpose, we evaluated gene and protein expression of inflammatory markers in tumor tissue, along with the profile of infiltrating macrophages in the tumor microenvironment. The first aspect examined was the expression of the tumor-derived factors described to take part in cachexia. Much to our surprise, it was actually the weight-stable group who presented higher values for lipid mobilizing factor (ZAG), while proteolysis inducing factor (PIF) was higher in cachectic patients. The literature provides evidence that these factors are present in cachexia, but no study, has to our knowledge, compared patients with matched tumor diagnosis with and without cachexia. Therefore, it is not impossible to speculate that tumor-derived factors actually have a role in inducing a better immune and metabolic regulatory response to the presence of the tumor. More studies are, nevertheless, required to further elucidate the importance of specific tumor-originated factors.

The microenvironment of solid tumors consists of tumor cells, infiltrating immune cells and matrix components (33, 34). In whole tumor tissue samples, we found higher TNF-α and CCL2 gene expression, along with higher CCL3 protein expression in cachectic patients, as compared to WSC. Billingsley et al. have reported similar results with *in vitro* studies in regard to TNF-α, IL-6, and leukemia inhibitory factor (LIF), in which co-culture of TNF-α with tumor cells augmented significantly cytokine production (35).

We have presently analyzed inflammation-related factors in whole tumor samples, having found that the pro-inflammatory cytokine IL-1β and the anti-inflammatory cytokine IL13 expression was altered (higher and lower, respectively) in cachectic cancer patients, as compared to WSC. The classical studies regarding tumor progression were initially driven to understand intrinsic changes in malignant cells (23). In the recent years, aspects related with the tumor microenvironment and to the host’s response to tumor progression have received more attention, and specially, the infiltrating immune cells, as their presence is associated with persistent inflammatory states (36, 37).

In order to establish whether tumors from cachectic patients and from WSC were different in terms of infiltration macrophage populations, we employed specific markers to identify macrophage sub-phenotypes. The results show fewer M2 macrophages in tumors of cachectic cancer patients, as compared with the weight stable group. Weber et al. demonstrated in patients with oral squamous cell carcinoma that increased polarization of macrophages toward a M2 phenotype is potentially correlated with a negative influence on tumor biology, resulting in more aggressive tumors (38). We failed to encounter studies in the literature that associate tumor infiltrating macrophage population with the presence of cachexia.

Considering that several inflammatory signaling pathways work in concert in promotion of inflammation, we performed Spearman’s correlation tests for tumor and subcutaneous adipose tissue data. The results show that CCL3 protein levels present a positive correlation with the expression of pro-inflammatory IL-1β protein in the patients’ tumors. In the subcutaneous adipose tissue, we report a positive correlation between CCL4 and TNF-β. These data corroborate the idea of complex and active interaction between the tumor and peripheral tissues, with major involvement of infiltrating immune cells.

The limitations of the study should be acknowledged. The previous body mass was informed by patients, and thus inaccuracies regarding this parameter are possible. Owing to human tissue sample implicit variation, some of the analyses were not performed with the total number of patients formerly enrolled, as some samples fell out of the detection range of the assays. The relative contribution of infiltrating monocytes for tissue inflammation was not assessed. Experiments with isolated cell populations are now being conducted.

## Conclusion

The results provide evidence that tumors from cachectic and weight stable cancer patients with same diagnosis show different secretory profile in regard to inflammatory factors and different macrophage phenotype percentage. An association between tumor-originated factors and adipose tissue inflammatory changes is proposed, as a positive correlation was found between tumor and adipose tissue-derived cytokines and inflammatory factors.

## Conflict of Interest Statement

The authors declare that the research was conducted in the absence of any commercial or financial relationships that could be construed as a potential conflict of interest.

## Supplementary Material

Figure S1 **Gating strategy for determination of macrophage-infiltrating subpopulations in tumor and adipose**. Specific gating strategies: **(A)** FSC-H vs FSC-A to exclude doublets. **(B)** FSC vs SSC to gate out the debris. **(C)** CD45+ to include all leukocytes. **(D)** CD14+ or CD11+ macrophages can be identified by markers such as CD14+ or CD11b+. **(E)** Unlabeled sample. **(F)** Labeled sample CCR7 (subpopulation M1), CXCR4+ (subpopulation M2), and double positive CCR7+ CXCR4+ (subpopulation M1–M2).
