# Supplementary figures and images for: Systemic Inflammation in Cachexia – Is Tumor Cytokine Expression Profile the Culprit?
Source: Front Immunol. 2015 Dec 24;6:629. doi: 10.3389/fimmu.2015.00629 (PMC4689790; doi:10.3389/fimmu.2015.00629)

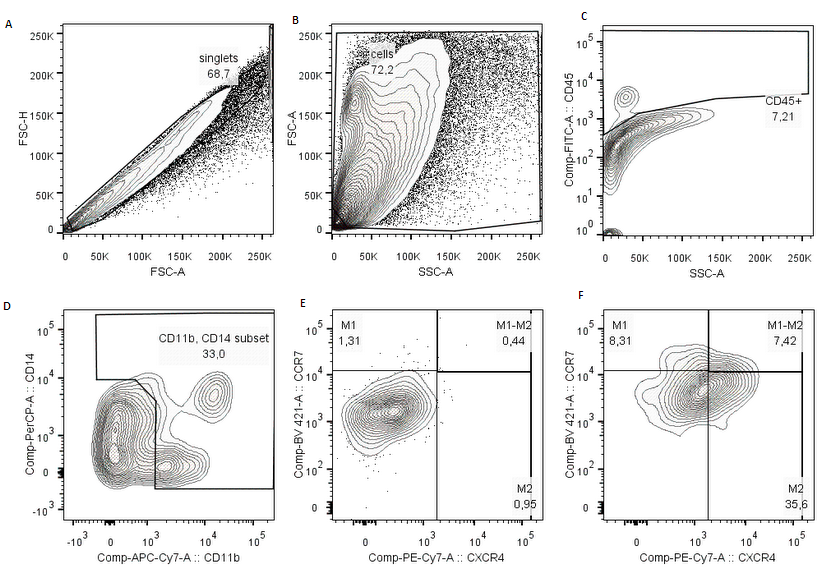

Supplement: Figure S1 — Gating strategy for determination of macrophage-infiltrating subpopulations in tumor and adipose. Specific gating strategies: (A) FSC-H vs FSC-A to exclude doublets. (B) FSC vs SSC to gate out the debris. (C) CD45+ to include all leukocytes. (D) CD14+ or CD11+ macrophages can be identified by markers such as CD14+ or CD11b+. (E) Unlabeled sample. (F) Labeled sample CCR7 (subpopulation M1), CXCR4+ (subpopulation M2), and double positive CCR7+ CXCR4+ (subpopulation M1–M2). [file image_1.tif]
